# Spermine Ameliorates DSS-Induced Ulcerative Colitis in Mice by Improving Mitophagy and Intestinal Microbiota

**DOI:** 10.3390/life16030417

**Published:** 2026-03-04

**Authors:** Ran Yu, Yamei Liu, Yating Zheng, Saisai Chen, Ling Tong, Jichun Wang, Chengmin Li, Chuanjian Zhang

**Affiliations:** 1 Jiangsu Key Laboratory of Sericultural Biology and Animal Biotechnology, School of Biotechnology, Jiangsu University of Science and Technology, Zhenjiang 212100, China; 18912250570@163.com; 2 National Research Center of Engineering and Technology for Veterinary Biologicals, Institute of Veterinary Immunology and Engineering, Jiangsu Academy of Agricultural Sciences, Nanjing 210014, China; lym613@hotmail.com (Y.L.); yt851110@163.com (Y.Z.); 20190981@jaas.ac.cn (S.C.); tl1994611@126.com (L.T.); jcwang@263.net (J.W.); 3 GuoTai (Taizhou) Center of Technology Innovation for Veterinary Biologicals, Taizhou 225300, China; 4 Jiangsu Co-Innovation Center for Prevention and Control of Important Animal Infectious Diseases and Zoonoses, Yangzhou 225009, China; 5 Jiangsu Key Laboratory for Food Quality and Safety-State Key Laboratory Cultivation Base of the Ministry of Science and Technology, Jiangsu Academy of Agricultural Sciences, Nanjing 210014, China

**Keywords:** spermine, ulcerative colitis, NLRP3, mitophagy, microbiota, mice

## Abstract

Spermine (Spe) plays a critical role in maintaining the integrity of the intestinal barrier and promoting intestinal development. However, the therapeutic role of Spe on ulcerative colitis (UC) remains unclear. This study aims to research the impact and mechanism of Spe on dextran sulfate sodium (DSS)-induced colitis in mice. Twenty-eight C57BL/6 mice were orally administered Spe before and during DSS treatment to evaluate its protective effects. Lipopolysaccharides (LPSs) were used to construct an in vitro UC model in IEC-6 cells. The study indicates that Spe treatment upregulated the expression of tight junction protein occludin and inhibited NLRP3 mediated inflammatory response by downregulating the levels of NLRP3, Caspase-1, IL-1β, IL-18 and TNF-α in the colon of DSS-treated mice. In addition, Spe enhanced mitophagy in colitis mice by increasing expressions of mitophagy factors (PINK1, Parkin, LC3-II) in DSS-treated mice. PINK1-mediated mitophagy helps alleviate LPS-induced mitochondrial damage in IEC-6 cells. Furthermore, Spe regulates the gut microbiota composition in mice with colitis by increasing the abundance of unclassified Muribaculaceae, reducing the levels of Firmicutes and Blautia, and lowering the Firmicutes/Bacteroidetes ratio. In conclusion, spermine exhibited treatment efficacy on DSS-induced colitis by inhibiting NLRP3-mediated inflammatory response, promoting mitophagy and improving intestinal microbial dysbiosis.

## 1. Introduction

Ulcerative colitis (UC), as a major inflammatory bowel disease, typically presents with symptoms including weight loss, abdominal pain, diarrhea, bloody stool, and other clinical symptoms and has gradually increased in the worldwide [1]. The inflammatory status and extended disease course increase the incidence of colorectal cancer in patients with UC [2]. Pathogenesis of UC is complex and unclear, including environment, genetic factors, psychological factors, mucosal immune imbalance, intestinal barrier damage, and gut microbiota dysbiosis [3,4]. Clinically, corticosteroids, immunomodulators, and anti-TNF agents are usually used for UC treatment [4]. However, these agents may cause some adverse side effects, coupled with high costs. Therefore, it is important to seek better substitutes for UC treatment.

The NLRP3 inflammasome plays a critical role in UC pathogenesis by driving IL-1β and IL-18 maturation and secretion [5]. Mitophagy is a selective autophagic process that removes damaged mitochondria and can negatively regulate the NLRP3 inflammasome [6]. DPTEN-induced kinase 1 (PINK1) is a central mediator of mitophagy, which recruits Parkin to accumulate on the mitochondrial surface, ultimately inducing mitophagy [6]. Promoting mitophagy may be a novel sight for treating UC by suppressing NLRP3 inflammasome activation.

Gut microbiota, as crucial components of the intestinal microenvironment, plays critical roles in maintaining intestinal homeostasis [7]. Imbalance of intestinal microbiota may promote gut inflammation, further inducing gut-related diseases, such as diarrhea, enterotoxaemia, and UC [8,9]. Current research indicates that there are significant differences in the composition of the gut microbiota between healthy individuals and patients with ulcerative colitis [10,11]. Regulation of gut microbiota has a beneficial impact on gut health, which has become a novel strategy for treating UC.

**Figure 1:**
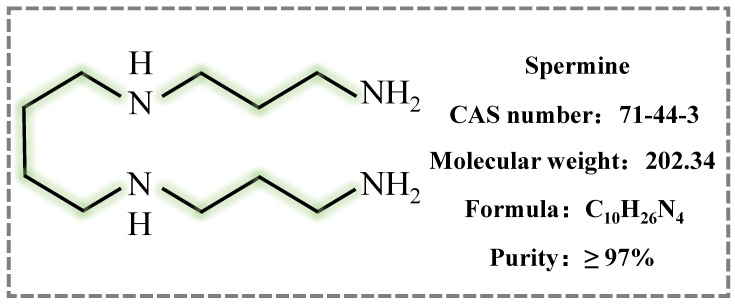
Structure of and information on spermine.

Spermine (Spe), a natural polyamine (Figure 1), is found in almost all tissues and cells. It is the terminal metabolic product of arginine through multiple enzymatic reactions. In the intestine of animal, which is obtained orally from exogenous dietary sources or produced by intestinal microbiota. Spe is involved in regulating various biological processes such as cell proliferation and differentiation, regulating immune response, improving the intestinal development, intestinal microbiota, intestinal barrier integrity, and growth performance [12,13,14]. Recent studies have further revealed that elevated intracellular spermine levels can directly block the assembly of the NLRP3 inflammasome and the activation of the downstream pyroptosis pathway by inhibiting potassium ion efflux [15]. Liu et al. found abnormalities in the arginine–polyamine metabolic axis in ulcerative colitis, specifically an accumulation of upstream arginine and a blockage in downstream spermidine synthesis [16]. This finding suggests that exogenous supplementation of spermidine to restore metabolic homeostasis may provide a basis for treating ulcerative colitis with spermidine. However, the protective effect of Spe on ulcerative colitis has not yet been reported in the literature. The potential mechanism also needs to be discussed.

This study investigated the anti-inflammatory effects of Spe by examining the expression of NLRP3 and pro-inflammatory cytokines, as well as the level of mitophagy. Using a DSS-induced mouse colitis model, it analyzed the regulatory effects of Spe on the gut microbiota, thereby elucidating its potential mechanism for the treatment of ulcerative colitis.

## 2. Materials and Methods

### 2.1. Network Pharmacology

Potential targets of Spermine were obtained from internet databases, including PubChem (https://pubchem.ncbi.nlm.nih.gov/, accessed on 20 June 2024) and the Comparative Toxicogenomics Database (https://ctdbase.org/, accessed on 20 June 2024). The targets related to colitis-associated diseases were screened from GeneCards (https://www.genecards.org/, accessed on 20 June 2024) and OMIM (https://www.omim.org/, accessed on 20 June 2024). Drug and disease gene names were standardized using the UniProt (https://www.uniprot.org/, accessed on 20 June 2024) database. This study screened the potential therapeutic targets of spermine in the treatment of colitis using the online Venn diagram tool (https://bioinformatics.psb.ugent.be/webtools/Venn/, accessed on 22 June 2024). STRING (https://cn.string-db.org/, accessed on 22 June 2024) database, with the confidence level set to the highest confidence threshold of “0.7”, was employed to construct a PPI network of overlapping targets and visualized using the PPI network in Cytoscape 3.9.1.

To further investigate the mechanism of spermine in treating colitis, the anti-colitis target genes of spermine were uploaded to the DAVID database (https://davidbioinformatics.nih.gov/, accessed on 25 June 2024). Functional annotation clustering was conducted for pathways with a *p*-value ≤ 0.05 and Benjamin value ≤ 0.05, and the top 25 pathways were selected. GO functional enrichment analysis was performed based on the GOTERM_BP_DIRECT, GOTERM_CC_DIRECT, and GOTERM_MF_DIRECT databases, and the results were visualized as bubble charts and bar charts using the bioinformatics platform (http://www.bioinformatics.com.cn/, accessed on 26 June 2024).

### 2.2. Animals and Experimental Design

This research protocol was approved by the Experimental Animal Ethics Committee of Jiangsu Academy of Agricultural Sciences (SYXK(Su) 2021-0073). Twenty-eight female C57BL/6 mice (SPF grade, weighing 26–28 g) were purchased from Nanjing Qinglongshan Animal Breeding Farm (Nanjing, China). The spermine used in the experiment was purchased from Shanghai Yuanye Bio-Technology Co., Ltd. (Yuanye, Shanghai, China). All of the mice were housed in the standard environment (temperature: 23–25 °C; humidity: 45–60%; light cycle: 12 h light/dark) with commercial mouse food and sterile water for 7 days. After that, all of the mice were classified into four different groups (*n* = 7) as follows: the control (Con) group, Spe group, dextran sulphate sodium (DSS) group, and DSS + Spe group. Mice were administered Spe orally at a dose of 0.4 mmol/g body weight (Spe and Spe + DSS groups) or sterile saline (Con and DSS groups) at days 3, 6, 9, and 12. The spermine dose (0.4 mmol/kg) was selected based on previously established safe and effective ranges in rodent studies [17,18]. From day 7 onwards, the drinking water of mice in the DSS group and Spe DSS group was supplemented with 3% DSS (Mw: 36,000–50,000 Da, MP Biomedicals, LLC, Irvine, CA, USA) for a duration of 7 days. At day 15, all mice were sacrificed, and serum sample and colonic tissue and content were rapidly collected. During the experiment, no deaths or obvious discomfort were observed in any of the groups of mice.

### 2.3. Evaluation of Colitis

As described previously [19], the daily body weight of the mice was recorded and the disease activity index score was calculated based on parameters such as stool characteristics, weight loss, and rectal bleeding. Then, the entire colon was collected and its length was measured. A 1 cm long section of the distal colon was taken, fixed in 4% paraformaldehyde, and then embedded in paraffin. The sample was cut into slices (5 µm thick) and hematoxylin and eosin (H&E) staining was performed. Pathological scoring was conducted according to methods described in the existing literature [19].

### 2.4. Inflammatory Cytokines in the Serum

Serum levels of inflammatory cytokines (IL-1β, IL-6, IL-18, and TNF-α) were measured using ELISA kits from Jiangsu Enzyme Immunoassay Co., Ltd. (Jiangsu Meimian Industrial Co., Ltd., Yancheng, China), strictly following the instructions provided in the manual.

### 2.5. Real-Time Quantitative PCR Analysis in Colonic Tissues

Total RNA was extracted from colon tissues using TRIzol reagent (TaKaRa, Otsu, Japan) as previously described [20] and was reverse-transcribed by a PrimeScript™ RT Master Mix Kit (TaKaRa, Otsu, Japan). Real-time quantitative PCR reactions were performed using SYBR-Green PCR premix (TaKaRa, Otsu, Japan) on a Roche LightCycler^®^ 480 system (Roche Diagnostics, Burgess Hill, UK). The relative expression level of the target gene was analyzed using the 2^−ΔΔCT^ method, with β-Actin used as an internal reference for normalization. The primer sequences used in this study are detailed in Table S1 [21,22,23].

### 2.6. Immunohistochemistry (IHC) Analysis

Colonic tissues from mice were fixed in 4% paraformaldehyde for 24 h, embedded in paraffin, and cut into 4 μm sections. Following deparaffinization and antigen retrieval, sections were blocked with 10% BSA for 1 h at room temperature. The sections were then incubated at 4 °C overnight with primary antibodies targeting NLRP3 (1:500, Proteintech, 30109-1-AP, Chicago, IL, USA), PINK1 (1:500, Proteintech, 23274-1-AP, Chicago, IL, USA), and LC3 (1:500, Proteintech, 14600-1-AP, Chicago, IL, USA). After washing, a fluorescent secondary antibody (1:200, Proteintech, SA00013-2, Chicago, IL, USA) was applied and incubated at room temperature for 50 min. Following another wash, DAB substrate was added and the slides were incubated in the dark at room temperature for 10 min. Finally, sections were counterstained with hematoxylin, and images were captured using a fluorescence microscope (Olympus, Tokyo, Japan).

### 2.7. Transmission Electron Microscopy (TEM)

We took 1 cm of distal colon tissue and fixed it sequentially with 2.5% glutaraldehyde and 2% osmium acid, followed by infiltration with acetone and epoxy resin. The tissue embedded in epoxy resin was sectioned into 70–90 nm thick slices and stained with uranyl acetate and lead citrate [24]. Autophagy and mitochondrion were observed under TEM.

### 2.8. Cell Culture and Treatment

IEC-6 cells were obtained from Servicebio (Wuhan, China) and maintained in DMEM high-glucose medium supplemented with 5–10% fetal bovine serum and 1% penicillin/streptomycin at 37 °C under 5% CO_2_. To assess the cytotoxicity of spermine, IEC-6 cells were treated with different concentrations of spermine (0, 2.5, 5, 7.5 μM) for 24 h, and cell viability was detected by the CCK-8 assay (Beyotime, Shanghai, China). For experimental treatments, cells were first incubated with 5 μM Spe for 24 h and subsequently exposed to 10 μM LPS for another 24 h. The concentrations of LPSs and Spe used were determined from prior dose optimization studies.

### 2.9. Cell Transfection

The siRNA targeting the PINK1 gene (siPINK1) and the negative control siRNA (siNC) were designed and synthesized by GenePharma Co., Ltd. (Shanghai, China). Transfection was performed using Lipofectamine 2000 (Invitrogen, Carlsbad, CA, USA), following the manufacturer’s protocol when the IEC-6 cells reached 60–70% confluence. The siRNA sequence used to interfere with PINK1 was 5′-GGACUCUCUUCCUCGUCAUTT-3′, and the antisense strand sequence was 5′-UAUCACAAGCUUCUGCUGCTT-3′. The sense strand sequence of siNC was 5′-UUCUCCGAACGUGUCACGUTT-3′, and the antisense strand sequence was 5′-ACGUGACACGUUCGGAGAATT-3′.

### 2.10. Measurement of Mitochondrial Membrane Potential (Δψm)

Mitochondrial membrane potential (Δψm) was measured using the JC-1 detection kit (Elabscience, Wuhan, China). According to the instructions of the kit, the IEC-6 cells cultured in the six-well plate were washed, JC-1 working solution was added, and incubated at 37 °C in the dark for 20 min. After washing, images were acquired using a fluorescence microscope (Olympus, Tokyo, Japan).

### 2.11. Detection of ROS Concentration (mtROS)

Mitochondrial reactive oxygen species levels were measured using the MitoSOX Red mitochondrial superoxide fluorescent probe (GlpBio Technology, San Diego, CA, USA). IEC-6 cells were incubated in a dark place at room temperature with 100 μL of 2.5 μM MitoSOX Red working solution for 20 min. After washing, images were acquired using a fluorescence microscope (Olympus, Tokyo, Japan).

### 2.12. Intestinal Microbiota Analysis

According to the instructions of the kit, bacterial DNA in colon contents was extracted using the E.Z.N.A.^®^ Soil DNA Kit (Omega Biotek, Norcross, GA, USA). PCR amplification of the V3-V4 region of the bacterial 16S rRNA gene using primer 338F 5′-ACTCCTACGGGAGGCAGCAG-3′ and 806R 5-GGACTACHVGG-TWTTAAT-3′. High-throughput sequencing was performed using the Illumina HiSeq 2500 platform (Beijing Biomarker Technologies company, Beijing, China). High-quality sequences were clustered into operational taxonomic units at a 97% similarity threshold. We used the Mothur software (v.1.48.0) to calculate alpha diversity indices (ACE, Chao1, Shannon, and Simpson) and performed principal coordinates analysis (PCoA) [25,26].

### 2.13. Statistical Analysis

Data were analyzed using the SPSS 20.0 software (SPSS Inc., Chicago, IL, USA). Data are expressed as mean ± SEM. The differences of bacterial abundance were analyzed using the Kruskal–Wallis test with false-discovery rate (FDR) adjustment. Differences of other data were analyzed using Turkey’s multiple comparisons test. *p* < 0.05 was considered as a statistically significant.

## 3. Results

### 3.1. Network Pharmacological Analysis

**Figure 2:**
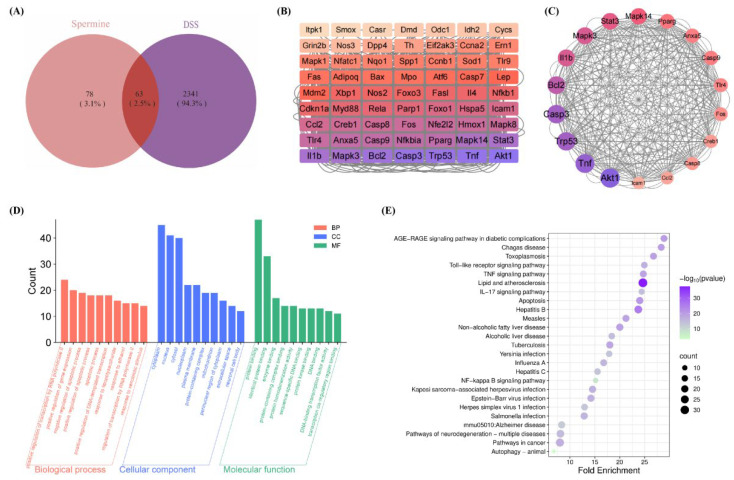
Network pharmacology. (**A**) Venn diagram of intersection target. (**B**) Protein interaction network of the common targets of spermine and colitis. (**C**) Core targets. (**D**) Gene ontology (GO) Functional Relationship Diagram. (**E**) KEGG pathway enrichment bubble plot.

After removing duplicates, 141 target genes for spe and 2404 targets for colitis were identified, with a total of 63 overlapping targets (Figure 2A). And the component–target interaction network was constructed using the Cytoscape 3.9.2 software (Figure 2B). Then, a PPI network was constructed to identify the key targets, which consists of 31 nodes and 349 edges (Figure 2C). The top 18 nodes were selected for visualization based on network topology, with node size reflecting connectivity (Table 1). Additionally, the top ten GO-BP, GO-MF, and GO-CC terms (Figure 2D) and the top 26 KEGG pathways (Figure 2E) were selected. The GO and KEGG enrichment analysis results indicate that the key targets of spermine in treating colitis are mainly involved in inflammation-related pathways and autophagy-related pathways.

### 3.2. Spermine Relieved DSS-Induced Colonic Injury in Mice

**Figure 3:**
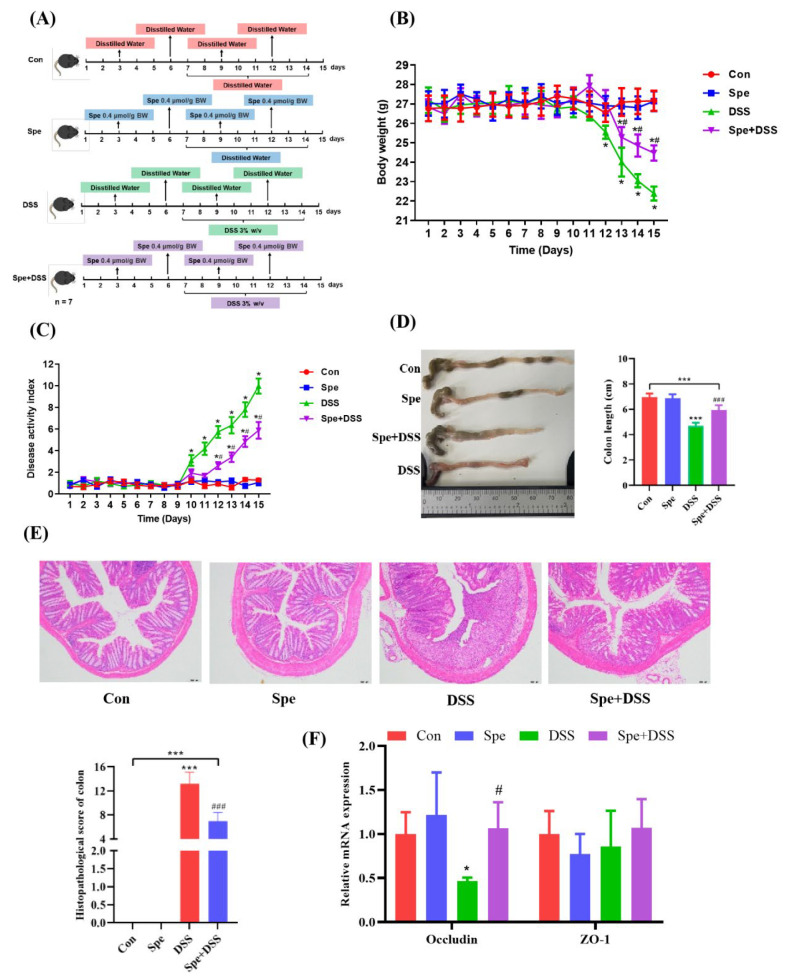
Spermine treatment alleviated DSS-induced colitis. (**A**) Experimental design of spermine intervention in a DSS-induced mouse model of colitis. (**B**) Body weight and (**C**) disease activity index (DAI) score were recorded daily for each group (*n* = 7). (**D**) The length of each colon group. (**E**) Representative H&E stained images of the colon (scale bar = 100 μm) and histopathological scores. (**F**) Relative mRNA expression levels of Occludin and ZO-1 genes in mouse colon tissues. Data are presented as mean ± SEM (* *p* < 0.05, *** *p* < 0.001 vs. control group. ^#^ *p* < 0.05, and ^###^ *p* < 0.001 vs. DSS group).

To further investigate the effects of Spe on ulcerative colitis, mice were administered Spe at a dose of 0.4 mmol/g body weight or an equivalent amount of sterile saline by gavage on days 3, 6, 9, and 12; except for the control group, 3% DSS was added to the drinking water of the remaining groups from day 7 to day 14 (Figure 3A). No obvious clinical symptoms were observed in the Spe group and control group. After oral administration of DSS, mice showed severe weight loss and the increased diarrhea with bloody and mucus stools compared to the control group (Figure 3B,C). When mice were treated with 3% DSS combined with Spe, we observed that weight loss and the DAI score were reduced (Figure 3B,C). Moreover, Spe treatment improved DSS-induced colon shortening (Figure 3D). The H&E staining results show that mice in the Spe group and control group had a normal colonic structure; DSS disrupted intestinal crypt structure and induced obvious inflammatory cell infiltration. The above results prove that the DSS-induced colitis model was successfully established. While Spe treatment relieved these symptoms, and the histological score also suggested that Spe treatment significantly improved colonic histological damage (Figure 3E). In addition, in terms of indicators related to mechanical barrier function (Occludin and ZO-1), DSS treatment significantly reduced the expression level of Occludin compared to the control group; the Spe treatment suppressed the decrease in Occludin (Figure 3F). These results indicate that Spe can relieve DSS-induced colonic injury in mice.

### 3.3. Spermine Modulated NLRP3-Mediated Inflammatory Response in DSS-Induced Colitis Mice

**Figure 4:**
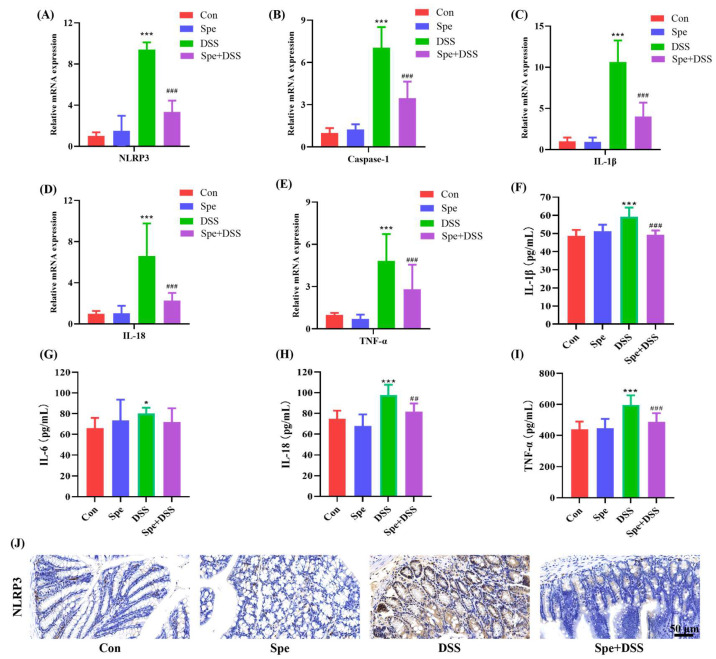
Spermine treatment inhibited NLRP3-mediated inflammatory response in DSS-treated mice. (**A**–**E**) Genes expressions related to NLRP3-mediated inflammatory response in the colon were measured. (**F**–**I**) The levels of pro-inflammatory cytokines in the serum. (**J**) Detection of NLRP3 inflammasome protein expression levels in the colon using immunohistochemistry. Data are presented as mean ± SEM (* *p* < 0.05, *** *p* < 0.001 vs. control group. ^##^ *p* < 0.01, and ^###^ *p* < 0.001 vs. DSS group).

In colonic tissues, DSS induces an NLRP3-mediated inflammatory response by upregulating the expression of NLRP3, Caspase-1, IL-1β, IL-18, and TNF-α, whereas Spe intervention significantly reduced the mRNA expression levels of these genes (Figure 4A–E). At the same time, the levels of pro-inflammatory cytokines (TNF-α, IL-1β, IL-6, and IL-18) in the serum of mice were increased after the DSS induction, while Spe treatment inhibited the change (Figure 4F–I). Consistent with the above results, the immunohistochemistry results show that Spe treatment can significantly reduce NLRP3 expression in colonic tissues of DSS-induced colitis mice (Figure 4J). These results suggest that Spe treatment could inhibit NLRP3-mediated inflammatory response in DSS-induced colitis mice.

### 3.4. Spermine Enhanced Mitophagy in DSS-Induced Colitis Mice

**Figure 5:**
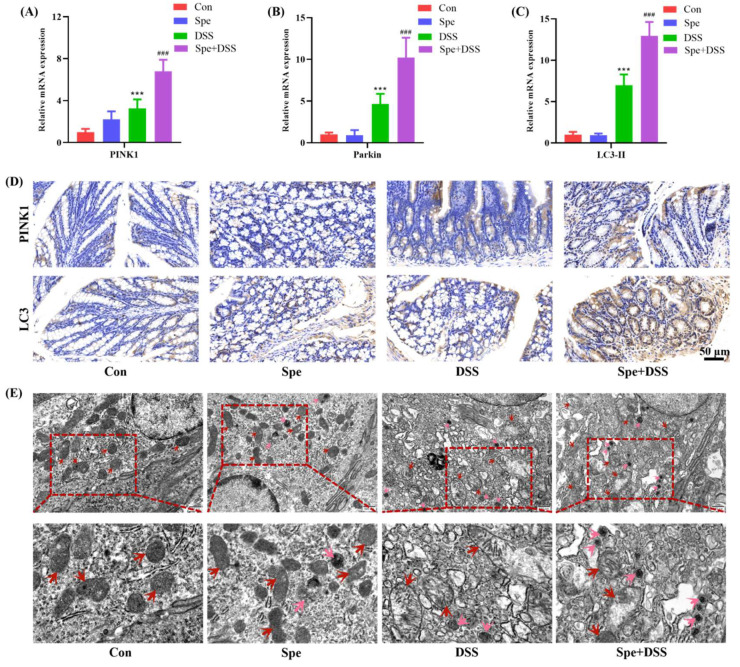
Spermine treatment enhanced mitophagy in DSS-treated mice. (**A**–**C**) Gene expressions related to mitophagy in the colon were measured. (**D**) The expression levels of PINK1 and LC3 in colon tissues detected by immunohistochemistry. (**E**) Ultrastructure of mitochondria and mitochondrial autophagosomes in the colon were assessed by transmission electron microscope TEM, the red arrows indicate abnormal mitochondrial structure, while the pink arrows represent autophagosomes (scale bars represent 0.5 μm). Data are presented as mean ± SEM. *** *p* < 0.001 vs. control group. ^###^ *p* < 0.001 vs. DSS group.

A previous study showed that mitophagy can negatively regulate NLRP3 inflammasomes; therefore, we analyzed the level of mitophagy-related genes. As shown in Figure 5A–C, compared with the control group, DSS treatment significantly upregulated the mRNA expression levels of PINK1, Parkin, and LC3-II. Spe treatment further increased the transcription levels of these genes. In agreement with the RT-qPCR findings, immunohistochemical analysis revealed that Spe administration further elevated the protein expression of PINK1 and LC3 in colonic tissues of mice with DSS-induced colitis (Figure 5D). Additionally, TEM analysis revealed that DSS induced mitochondrial structure damage. Relative to the DSS-treated group, Spe intervention led to a significant decrease in the quantity of impaired mitochondria, accompanied by a notable increase in autophagosome numbers (Figure 5E). These results suggest that Spe treatment further activated mitophagy in DSS-induced colitis.

### 3.5. Spe on IEC-6 Cytotoxicity Evaluation

**Figure 6:**
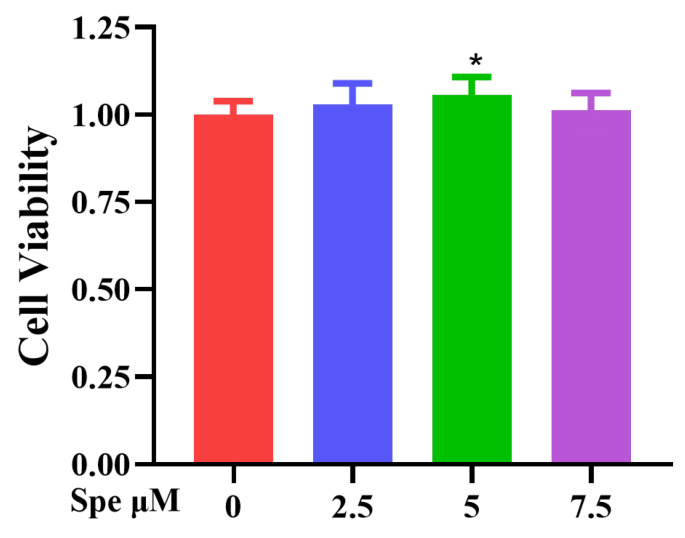
Spe cytotoxicity evaluation on IEC-6 cells. The effects of different concentrations of Spe (0, 2.5, 5, 7.5 μM) on IEC-6 cell viability were assessed using the CCK-8 assay. Data are presented as mean ± SEM. * *p* < 0.05 vs. control group.

The effect of different concentrations of spermidine (0, 2.5, 5, 7.5 μM) on the viability of IEC-6 cells after 24 h treatment was detected by the CCK-8 method. The results show that 5 μM spermidine significantly enhanced cell viability, while 7.5 μM spermidine caused a decrease in cell viability (Figure 6). Based on this, 5 μM was selected as the treatment concentration of spermidine for the subsequent experiments.

### 3.6. PINK1-Mediated Mitophagy Contributed to the Mitochondrial Protective Effects of Spe in LPS-Stimulated IEC-6 Cells

**Figure 7:**
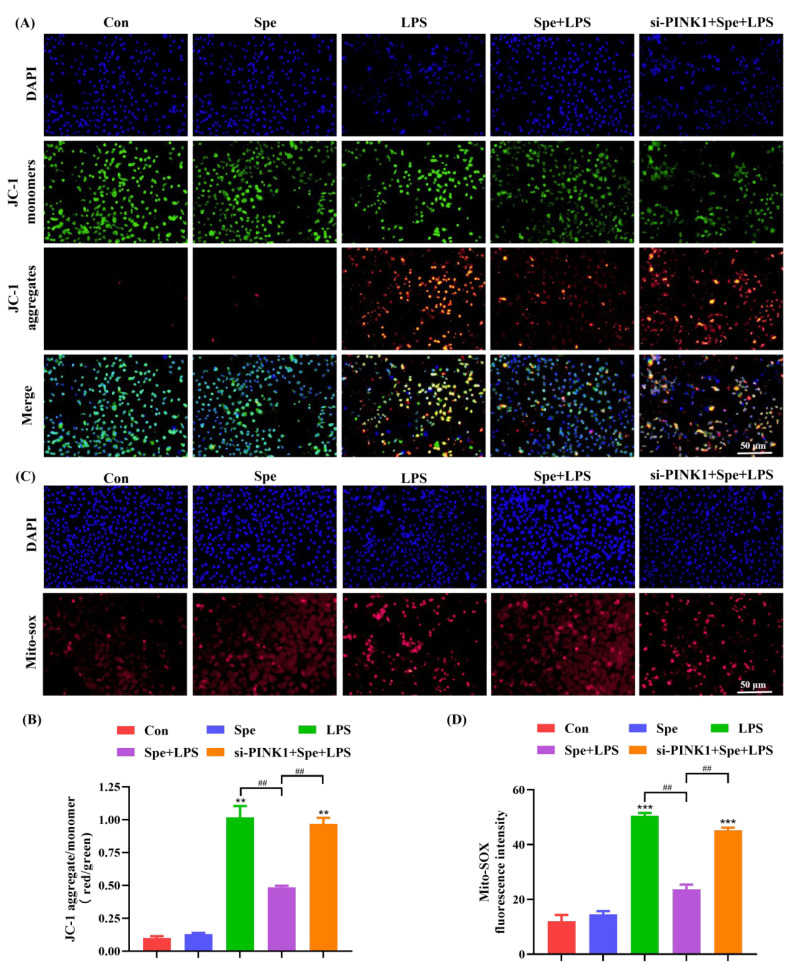
Effects of mitophagy inhibition on LPS-induced mitochondrial damage in IEC-6 cells. (**A**,**B**) Mitochondrial membrane potential was detected using the JC-1 staining method. (**C**,**D**) Assessment of mitochondrial reactive oxygen species levels. Data are presented as mean ± SEM. ** *p* < 0.01, *** *p* < 0.001 vs. control group. ^##^ *p* < 0.01 vs. DSS group.

Given that mitophagy negatively regulates NLRP3 inflammasome activation, mainly by effectively removing damaged mitochondria and thereby preventing the continuous accumulation of mitochondrial damage signals, we further explored the effect of Spe on LPS-induced mitochondrial damage. The results indicate that, compared with the control group, LPS treatment significantly increased the fluorescence intensity of JC-1 aggregates in IEC-6 cells (reflecting the level of mitochondrial membrane potential), whereas the Spe treatment markedly inhibited this phenomenon. However, after silencing PINK1 to inhibit mitophagy, the fluorescence intensity of JC-1 aggregates significantly increased again (Figure 7A,B). Consistently, the Spe treatment effectively reduced ROS accumulation in IEC-6 cells, whereas PINK1 knockdown reversed this protective effect (Figure 7C,D). These results suggest that inhibiting mitophagy through PINK1 knockdown can counteract the protective effect of Spe on mitochondrial function.

### 3.7. Spermine Modulated Gut Microbiota in DSS-Induced Colitis Mice

**Figure 8:**
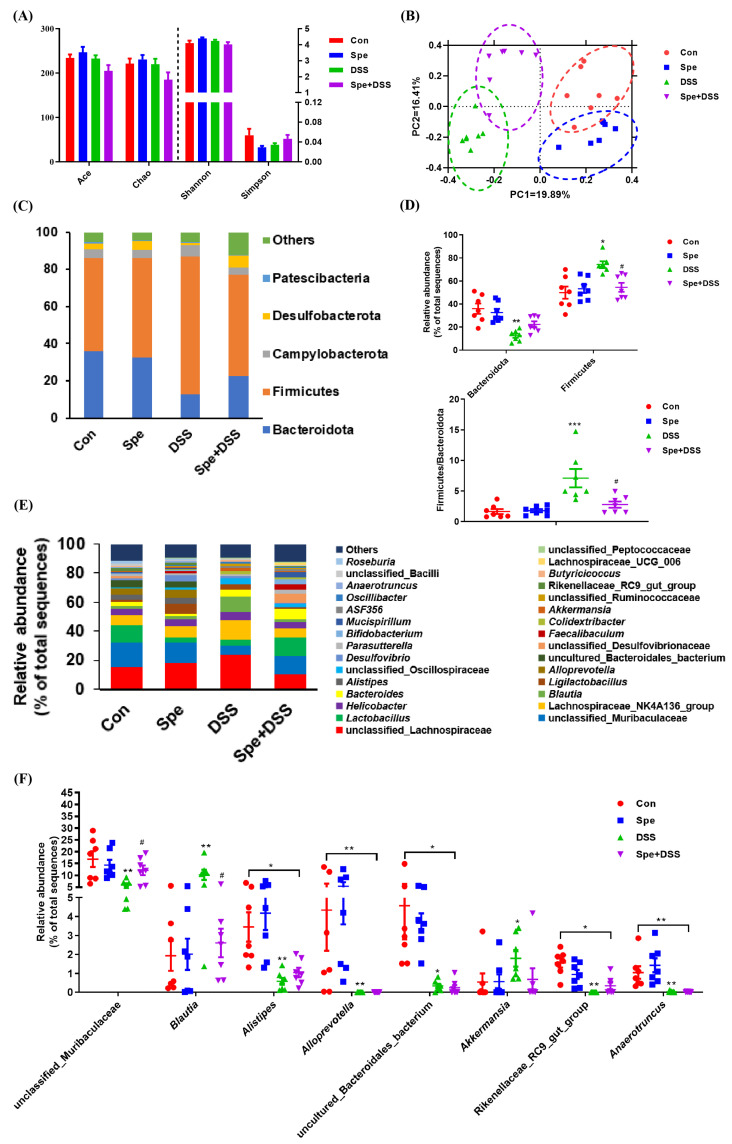
Spermine treatment improved intestinal microbiota in DSS-treated mice. Mouse colon contents for DNA extraction and 16S rRNA gene sequencing were collected. (**A**) Alpha diversity indices. (**B**) PCoA analysis based on Bray–Curtis distance. (**C**) Microbial community structure at the phylum taxonomic level. (**D**) Differences in the microbial communities of the portal vein. (**E**) Microbial composition at the genus level. (**F**) Differences in horizontal microbial communities. Data are presented as mean ± SEM. * *p* < 0.05, ** *p* < 0.01; *** *p* < 0.001 vs. control group. ^#^ *p* < 0.05 vs. DSS group.

The 16S rRNA was sequenced using the Illumina MiSeq platform to analyze the composition of bacterial communities. The bacterial richness and diversity indices are shown in Figure 8A,B. The species richness indices (ACE and Chao indices) and the diversity indices (Simpson and Shannon indices) did not show significant differences among the four groups. The principal coordinate analysis (PCoA) based on Bray–Curtis distances demonstrated a clear community separation among the four sample groups.

At the phylum level, Firmicutes (58.02%) and Bacteroidetes (25.89%) were the two major phyla. Campylobacterota and Desulfobacterota constituted the next two phyla, accounting for 4.89% and 23.94% (Figure 8C). Compared with the control group, the relative abundance of Bacteroidetes was significantly reduced in the DSS group (Figure 8D). DSS treatment significantly increased the abundance of Firmicutes, whereas the Spe intervention inhibited this increase (Figure 8D). The Firmicutes/Bacteroidota ratio was increased in the DSS group but decreased by the Spe treatment (Figure 8D).

The top 30 bacterial genera in terms of abundance in the ileum and colon are shown in Figure 8E. The 10 most predominant genera were unclassified_Lachnospiraceae, unclassified_Muribaculaceae, *Lactobacillus*, Lachnospiraceae_NK4A136_group, *Helicobacter*, *Blautia*, *Bacteroides*, *Ligilactobacillus*, *Alistipes*, and *Alloprevotella*. A decrease in the abundance of *Alistipes*, *Alloprevotella*, uncultured_Bacteroidales_bacterium, Rikenellaceae_RC9_gut_group, and *Anaerotruncus* by DSS but failed to be recovered by the Spe treatment (Figure 8F). Furthermore, DSS treatment caused an increase in *Akkermansia*. The Spe treatment restored unclassified_Muribaculaceae and *Blautia* to levels comparable with healthy controls in DSS-exposed mice (Figure 8F). Taken together, these results indicate that the Spe intervention can modulate the gut microbiota in DSS-induced colitis mice.

### 3.8. Correlations Between Gut Microbiota and Functional Genes in DSS-Induced Colitis Mice

**Figure 9:**
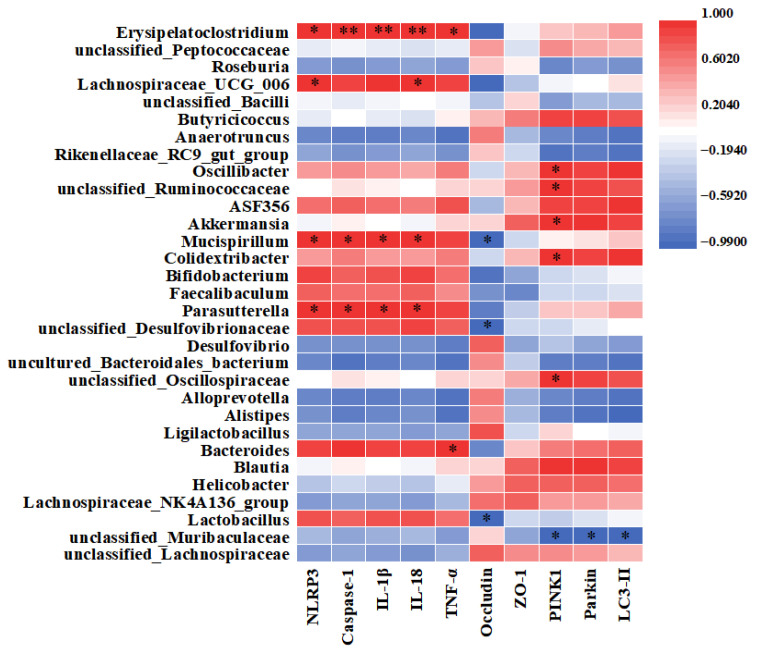
The correlation analysis using Spearman’s method was conducted between different bacteria and clinical indicators or functional genes of DSS-induced colitis in mice. * *p* < 0.05, ** *p* < 0.01.

A Spearman rank correlation analysis was conducted to assess the relationship between the relative abundance of gut microbiota in DSS-induced colitis mice and clinical indicators (Occludin, ZO-1) as well as functional genes, including inflammation-related genes (NLRP3, Caspase-1, IL-1β, IL-18, TNF-α) and mitophagy-related genes (PINK1, Parkin, LC3-II). Results show that *Erysipelatoclostridium*, *Mucispirillum*, and *Parasutterella* were positively correlated with NLRP3 inflammasome genes. *Mucispirillum*, *unclassified_Desulfovibrionaceae*, and *Lactobacillus* were negatively correlated with Occludin. In addition, *Oscillibacter*, *unclassified_Ruminococcaceae*, *Akkermansia*, *Colidextribacter*, and *unclassified_Oscillospiraceae* were positively correlated with PINK1. Moreover, *unclassified_Muribaculaceae* showed a negative correlation with mitophagy-related genes (Figure 9).

## 4. Discussion

The incidence of UC is continuously increasing worldwide and brings pain and economic pressure to the patients [27]. Previous studies showed that Spe regulates immune response and improves intestinal microbiota and intestinal barrier integrity [13,14]. It is unclear whether Spe has a therapeutic effect on UC; its underlying mechanism also needs to be elucidated. Network pharmacology analysis showed that key targets of spermine in colitis were primarily associated with inflammation and autophagy-related pathways. Moreover, the experimental study has confirmed for the first time Spe exert therapeutic effects on UC by regulating the dysregulated gut microbiota, inhibiting NLRP3-mediated inflammatory response and promoting mitophagy.

DSS is often used to establish mouse colitis models. In the present investigation, mice with DSS-induced colitis exhibited clinical manifestations including reduced body weight, diarrheal symptoms, and fecal blood presence. However, the Spe treatment improved body weight loss, colon length shortening, and DAI. H&E staining revealed reduced inflammatory infiltration in the colon tissues of mice treated with both Spe and DSS. When UC occurs, inflammatory cytokines are secreted and involved in inflammatory cascades [28]. The current investigation measured the expression of pro-inflammatory cytokine, which was elevated in the colon of mice with DSS-induced colitis, while these pro-inflammatory cytokines were reduced after the Spe treatment. A previous study has showed the reciprocal regulation between colonic immunity and systemic immunity [29]. This suggests that changes in pro-inflammatory cytokines within the colon may be partially responsible of the corresponding alterations in serum levels. As a key component of the mechanical barrier, tight junction proteins can effectively prevent harmful substances in the intestine from entering the bloodstream. The results of this study indicate that DSS-induced ulcerative colitis in mice significantly reduces the mRNA expression of occludin in the colon, whereas this expression is markedly restored following the Spe intervention. The results suggest that the remission effect of Spe on ulcerative colitis may be attributed to the downregulation of pro-inflammatory cytokines and the upregulation of occludin mRNA expression.

NLRP3 inflammasome activation is a key event in DSS-induced colitis [30,31]. Consistent with this, we found that the mRNA expression levels of NLRP3 and downstream pro-inflammatory cytokines (IL-1β, IL-18, TNF-α) and caspase-1 were significantly elevated in the colons of DSS-treated mice, whereas this expression was effectively suppressed following the Spe intervention. Our research findings indicate that Spe may exert an anti-ulcerative colitis effect by inhibiting NLRP3-mediated inflammatory responses. Mitochondrial dysfunction serves as the upstream signal for the activation of NLRP3 inflammasome, while mitochondrial autophagy, by eliminating damaged mitochondria, plays a negative regulatory role in this process [32]. The research conducted by Xu et al. indicates that spermine alleviates inflammation by enhancing autophagic activity [33,34]. The findings of this study demonstrate that Spe increased the number of mitophagy vesicles and expressions of mitophagy factors (PINK1, Parkin, LC3-II) in mice with DSS-induced colitis. At the same time, knocking down PINK1, which inhibits mitophagy, reversed the protective effect of Spe on mitochondrial function. Therefore, we speculate that Spe inhibits NLRP3-mediated inflammatory response probably by enhancing PINK1/Parkin-mediated mitophagy. It is worth noting that recent studies have shown that spermine inhibits the expression of GBP5 in the EV71 infection model, thereby blocking the assembly of the NLRP3 inflammasome and the release of downstream inflammatory factors [35]. This finding is partially consistent with the results of this study, which demonstrated that spermine inhibits the expression of NLRP3 in colonic tissues. Thus, more in vitro studies in the future are necessary to clear whether Spe alleviated NLRP3-mediated inflammatory response via PINK1/Parkin-mediated mitophagy.

Gut microbiota dysbiosis is a hallmark of UC [13]. In this study, the Spe intervention reversed DSS-induced alterations in microbial composition, including a reduction in the Firmicutes/Bacteroidetes ratio, suppression of Blautia enrichment, and restoration of Muribaculaceae abundance. Muribaculaceae are major producers of short-chain fatty acids (SCFAs), which support intestinal barrier function by serving as energy substrates for colonocytes and enhancing tight junction integrity [36]. The recovery of Muribaculaceae by Spe may thus contribute to the observed upregulation of occludin expression and improvement of barrier function. Blautia, which was increased by DSS and suppressed by Spe, has been associated with altered tryptophan metabolism in IBD. Tryptophan-derived metabolites activate aryl hydrocarbon receptor (AhR) signaling, which plays a protective role in intestinal immunity [37]. The Spe-induced reduction in Blautia may help rebalance AhR-mediated mucosal immune responses. Interestingly, Li et al. recently identified an arginine–agmatine–spermidine biosynthesis pathway in gut commensals and demonstrated that upregulation of polyamine synthesis genes in Bacteroides correlates with elevated fecal polyamines in IBD patients [38]. This finding, together with our observation that exogenous spermine restores Muribaculaceae abundance and alleviates colitis, highlights a bidirectional host–microbe polyamine metabolic axis: while aberrant bacterial polyamine overproduction may reflect dysbiosis, exogenous polyamine intervention can restore metabolic equilibrium and support beneficial taxa. Although this bidirectional metabolic axis provides a conceptual framework, the exact mechanism by which Spe exerts selective effects on different bacterial groups remains to be elucidated.

The observed reduction in Blautia by Spe may involve direct antimicrobial effects against specific strains, as spermine has been shown to inhibit potential pathogens such as *H. pylori* and *E. coli* in vitro [39,40]. In contrast, the restoration of Muribaculaceae abundance is more likely attributable to metabolic niche remodeling—spermine may serve as a nitrogen/carbon source for these SCFA-producing bacteria, and its barrier-protective effects create a favorable anaerobic environment for their expansion [41,42]. This bidirectional model is supported by recent studies demonstrating that beneficial taxa such as *Akkermansia muciniphila* produce polyamine derivatives (e.g., N-acetylspermidine) that enhance barrier function [41], while pathogenic Salmonella exploit host polyamines for oxidative stress resistance [43,44]. These findings highlight the strain-specific nature of polyamine–microbiota interactions and suggest that Spe’s effects are not simply broad-spectrum antimicrobial but involve complex host–microbe metabolic crosstalk. Future studies using co-culture systems, fecal microbiota transplantation, and metagenomics are warranted to establish causality and elucidate strain-level mechanisms.

This study has several limitations. At the mechanism level, although the in vitro PINK1 knockdown experiments indicated that mitochondrial autophagy was involved in the protective effect of spermidine, the lack of in vivo functional deficiency studies (such as PINK1 knockout or fecal microbiota transplantation) limited our ability to infer the causal role of mitochondrial autophagy and microbiota regulation in the therapeutic effect of spermidine. Furthermore, the protein levels of key inflammatory mediators are inadequately verified-NLRP3 is only detected through immunohistochemistry, while Caspase-1 is only detected at the mRNA level, neither of which achieves the quantitative accuracy of Western blot. At the same time, we did not investigate whether the observed effects were mediated by spermidine itself or were achieved through the metabolism of SMOX to produce spermine, nor did we measure the levels of colonic polyamines or the activity of SMOX [39]. In conclusion, the aforementioned deficiencies at the mechanism level suggest that, in the future, it is necessary to combine gene knockout animal models, metabolomics, and clinical dosage exploration studies to further clarify the mechanism of spermidine in treating UC and its potential for transformation.

## 5. Conclusions

**Figure 10:**
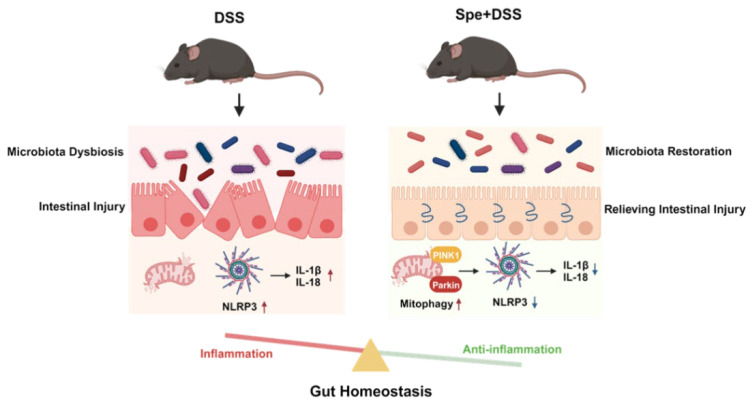
Potential mechanism of Spermine treatment alleviating DSS-induced colitis. First, spermidine enhances mitochondrial autophagy by upregulating the expression of PINK1/Parkin, thereby eliminating damaged mitochondria and reducing the production of reactive oxygen species. This, in turn, inhibits the activation of the NLRP3 inflammasome and the subsequent release of IL-1β/IL-18, thereby alleviating the inflammatory response. Secondly, spermidine can reverse the intestinal flora imbalance induced by DSS, manifested as a decrease in the ratio of Firmicutes to Bacteroidetes, inhibition of Blautia enrichment, and restoration of the abundance of beneficial Muribaculaceae. These two pathways work together in synergy, jointly forming an integrated mechanism by which spermidine inhibits the NLRP3-mediated inflammatory response, promotes mitochondrial autophagy, and improves intestinal flora imbalance to alleviate DSS-induced colitis.

This study demonstrates that Spe can effectively alleviate DSS-induced colitis, and its mechanism of action may involve the following two aspects (Figure 10): the first one is involved the regulation of the gut microbiota; the second one is associated with the improvement of NLRP3-mediated inflammatory response and induction of PINK1/Parkin-mediated mitophagy.

## Figures and Tables

**Table 1:** Top 18 core targets.

Target	Degree	Betweenness unDir	Closeness unDir
Akt1	116	338.15890	0.01515
Tnf	114	145.80941	0.01493
Trp53	112	160.71235	0.01471
Casp3	112	117.29512	0.01471
Bcl2	108	96.55591	0.01429
Mapk3	104	105.50908	0.01389
Il1b	104	79.74973	0.01389
Stat3	102	65.92444	0.01370
Mapk14	100	51.36379	0.01351
Pparg	92	45.56130	0.01282
Anxa5	90	31.71610	0.01266
Casp9	90	42.24245	0.01266
Tlr4	88	33.09896	0.01250
Fos	86	57.89875	0.01235
Creb1	84	33.20073	0.01220
Casp8	84	32.18548	0.01220
Ccl2	82	126.30812	0.01205
Icam1	80	35.74752	0.01190

## Data Availability

The sequence information was submitted to the GenBank Sequence Read Archive database with the Bioproject accession number PRJNA1005778. No additional new data were created, or all other data supporting the reported results are available within the article and its Supplementary Materials.

## Supplementary Materials

The following supporting information can be downloaded at: https://www.mdpi.com/article/10.3390/life16030417/s1. Table S1. Primers for quantitative real-time PCR.

## Abbreviations

The following abbreviations are used in this manuscript:

UC	Ulcerative colitis
DSS	Dextran sulfate sodium
Spe	Spermine
LPS	Lipopolysaccharide
NLRP3	Nod-like receptor protein 3
PINK1	DPTEN-induced kinase 1
IL-1β	Interleukin-1β
DAI	Disease activity index
FDR	False-discovery rate
GO-BP	Biological process
GO-MF	Molecular function
GO-CC	Cellular component
PCoA	Principal coordinate analysis
