# Supplementary material for: Spermine Ameliorates DSS-Induced Ulcerative Colitis in Mice by Improving Mitophagy and Intestinal Microbiota
Source: Life (Basel). 2026 Mar 4;16(3):417. doi: 10.3390/life16030417 (PMC13027953; doi:10.3390/life16030417)
Supplement: Supplementary file 1 [file life-16-00417-s001.zip › life-4157171-supplementary.pdf]

Table S1: Primers for quantitative real-time PCR

Primers	Primer sequences (5'-3')	Reference	Annealing temp.(°C)
Beta actin F	GGCTGTATTCCCCTCCATCG	[21]	60
Beta actin R	CCAGTTGGTAACAATGCCATGT		
IL-1β F	GCAACTGTTCCTGAACTCAACT	[22]	60
IL-1β R	ATCTTTTGGGGTCCGTCAACT		
IL-6 F	CTGCAAGAGACTTCCATCCAG	[22]	60
IL-6 R	GAGGGAATGCCCGTGGACGG		
IL-18 F	GACTCTTGCGTCAACTTCAAGG	[21]	60
IL-18 R	CAGGCTGTCTTTTGTCAACGA		
TNF-α F	CAGGCGGTGCCTATGTCTC	[22]	60
TNF-α R	CGATCACCCCGAAGTTCAGTAG		
NLRP3 F	GTGGTGACCCTCTGTGAGGT	[22]	60
NLRP3 R	TCTTCCTGGAGCGCTTCTAA		
Caspase-1 F	TATCCAGGAGGGAATATGTG	[22]	60
Caspase-1 R	ACAACACCACTCCTTGTTTC		
Occludin F	TGAAAGTCCACCTCCTTACAGA	[22]	60
Occludin R	CCGGATAAAAAGAGTACGCTGG		
ZO-1 F	GCCGCTAAGAGCACAGCAA	[22]	60
ZO-1 R	GCCCTCCTTTTAACACATCAGA		
PINK1 F	TTCTTCCGCCAGTCGGTAG	[23]	60
PINK1 R	CTGCTTCTCCTCGATCAGCC		
Parkin F	GAGGTCGATTCTGACACCAGC	[23]	60
Parkin R	CCGGCAAAAATCACACGCAG		
LC3-II F	TTATAGAGCGATACAAGGGGGAG	[23]	60
LC3-II R	CGCCGTCTGATTATCTTGATGAG		

[1]**Table S1:** Primers for quantitative real-time PCR

References:

21. Wan, Y.; Xu, L.; Wang, Y.; Tuerdi, N.; Ye, M.; Qi, R., Preventive effects of astragaloside iv and its active sapogenin cycloastragenol on cardiac fibrosis of mice by inhibiting the nlrp3 inflammasome. *Eur J Pharmacol* **2018**, *833*, 545-554.
22. Zhou, X.; Zhang, B.; Zhao, X.; Lin, Y.; Wang, J.; Wang, X.; Hu, N.; Wang, S., Chlorogenic acid supplementation ameliorates hyperuricemia, relieves renal inflammation, and modulates intestinal homeostasis. *Food Funct* **2021**, *12*, 5637-5649.
23. Zhang, J.; Tang, L.L.; Li, L.Y.; Cui, S.W.; Jin, S.; Chen, H.Z.; Yang, W.M.; Xie, D.J.; Yu, G.R., Gandouling tablets inhibit excessive mitophagy in toxic milk (tx) model mouse of wilson disease via pink1/parkin pathway. *Evid Based Complement Alternat Med* **2020**, *2020*, 3183714.
